# Multivariate classification of Brugada syndrome patients based on autonomic response to exercise testing

**DOI:** 10.1371/journal.pone.0197367

**Published:** 2018-05-15

**Authors:** Mireia Calvo, Daniel Romero, Virginie Le Rolle, Nathalie Béhar, Pedro Gomis, Philippe Mabo, Alfredo I. Hernández

**Affiliations:** 1 Univ Rennes, CHU Rennes, Inserm, LTSI – UMR 1099, Rennes, France; 2 Dept. ESAII, CREB, Universitat Politècnica de Catalunya, Barcelona, Spain; 3 CIBER of Bioengineering, Biomaterials and Nanomedicine, Zaragoza, Spain; University of Minnesota, UNITED STATES

## Abstract

Ventricular arrhythmias in Brugada syndrome (BS) typically occur at rest and especially during sleep, suggesting that changes in the autonomic modulation may play an important role in arrhythmogenesis. The autonomic response to exercise and subsequent recovery was evaluated on 105 patients diagnosed with BS (twenty-four were symptomatic), by means of a time-frequency heart rate variability (HRV) analysis, so as to propose a novel predictive model capable of distinguishing symptomatic and asymptomatic BS populations. During incremental exercise, symptomatic patients showed higher *HF*_*nu*_ values, probably related to an increased parasympathetic modulation, with respect to asymptomatic subjects. In addition, those extracted HRV features best distinguishing between populations were selected using a two-step feature selection approach, so as to build a linear discriminant analysis (LDA) classifier. The final features subset included one third of the total amount of extracted autonomic markers, mostly acquired during incremental exercise and active recovery, thus evidencing the relevance of these test segments in BS patients classification. The derived predictive model showed an improved performance with respect to previous works in the field (*AUC* = 0.92 ± 0.01; *Se* = 0.91 ± 0.06; *Sp* = 0.90 ± 0.05). Therefore, based on these findings, some of the analyzed HRV markers and the proposed model could be useful for risk stratification in Brugada syndrome.

## Introduction

Brugada syndrome (BS) is an inherited disease presenting a typical pattern on the electrocardiogram (ECG), characterized by a distinct ST-segment elevation in right precordial leads, associated with a high risk for unexpected sudden cardiac death (SCD), secondary to ventricular fibrillation (VF) in absence of any apparent structural cardiopathy [1, 2]. Since its initial description in 1992 as a new cardiac syndrome [3], BS has raised a great interest due to its high incidence, especially in far eastern countries, and its association with sudden death in young adults and, less frequently, in infants and children. It has been estimated that BS is responsible for 4–12% of the total amount of SCD and for 20% of SCD in patients with structurally normal hearts [4, 5].

Although several methods have been evaluated for the prediction of VF occurrence, studies based on the largest clinical series including BS patients only proved two consistent and reliable predictors of major cardiac events: documented symptoms and spontaneous type 1 Brugada-like ECG pattern [5, 6]. As a consequence, risk stratification in order to determine the best treatment approach for these patients still remains challenging, especially for asymptomatic individuals without documented VF episodes.

Ventricular arrhythmias (VA) in BS typically occur at rest and especially during sleep, suggesting that parasympathetic activity may play a relevant role in the arrhythmogenesis of the disease [7, 8]. Moreover, a sympathetic autonomic dysfunction on BS patients has been reported in previous works on cardiac autonomic nervous system (ANS) analysis based on positron emission tomography [8–11]. Thus, changes in the autonomic modulation captured by heart rate variability (HRV) analysis may provide useful information for the prediction of VA in these patients. Indeed, the autonomic function has already been studied in BS, but most previously reported autonomic markers are based on long-term measurements, being time-consuming and leading to contradictory results [12–18]. The evaluation of the autonomic response can be better characterized by stimulating the ANS in a controlled and repeatable fashion, by applying standardized maneuvers such as physical stress testing, pharmacological stimulations or the head-up tilt test. Physical exercise induces an increase in sympathetic activity and a parasympathetic withdrawal, resulting in higher heart rates (HR). Conversely, post-exercise cardio-deceleration is mediated by a progressive rise in vagal activity [19], as well as a continued sympathetic recession [20]. Indeed, previous works have reported the potential of exercise testing to predict VA in patients suffering from BS [21–25]. Nevertheless, we are not aware of any study having analyzed the temporal progression of the autonomic response under conditions of exercise and recovery in this population.

In this work, a time-frequency approach was applied on 105 BS patients at different levels of risk (symptomatic and asymptomatic) so as to characterize the temporal evolution of several HRV features in response to exercise. Then, a multivariate approach based on a step-based machine learning method was implemented to identify those extracted HRV features best distinguishing between populations. Based on the hypothesis that changes in the autonomic function could improve prognosis interpretation in BS, the main objective of the study was to build a multivariate classifier capable of identifying patients at high risk.

## Materials and methods

### Study population

The standard 12-lead ECG recordings from 118 consecutive patients diagnosed with Brugada syndrome who took part in a physical stress test were collected during a prospective, multicentric study conducted between 2009 and 2013 in the Cardiology department of the Rennes University Hospital (CHU de Rennes), in France. Participants were enrolled in 6 French hospitals located in Rennes, Saint Pierre de la Réunion, Nantes, Bordeaux, Brest and La Rochelle. The study protocol was approved by the respective local ethics committees: Comité d’Éthique du CHU de Rennes, Comité d’Éthique du CHU Saint-Pierre, Comité d’Éthique du CHU de Nantes, Comité d’Éthique du CHU de Bordeaux, Comité d’Éthique du CHU de Brest and Comité d’Éthique du Centre Hospitalier de La Rochelle. All patients provided their written informed consent before participation. Nevertheless, only 105 recordings met the quality criteria to be included in the analysis.

In accordance with the current guidelines [1, 2], BS was diagnosed when a coved ST-segment elevation (≥ 0.2 mV) was recorded in at least one right precordial lead (V1 and/or V2) located in the 2*^nd^*, 3*^rd^* or 4*^th^* intercostal space, in the presence or absence of sodium-channel-blocking agent.

In order to characterize populations with different levels of risk, patients were classified as symptomatic and asymptomatic, based on their medical history. Twenty-four patients presented documented symptoms of ventricular origin: syncope (50%), cardiac arrest (41.7%), dizziness (12.5%) and, less frequently, palpitations and nocturnal convulsions (4.2%). The remaining 81 patients were considered as asymptomatic.

Participant ages ranged from 19 to 74 years old (45.17 ± 13.62 years old) and 76.2% were males. ICDs had been implanted in 18 of 81 (22.2%) asymptomatic patients, based on a positive EPS (Electrophysiological Study) test, whereas all symptomatic patients had ICDs implanted. Among 76 patients (19 were symptomatic) in whom genetic analysis was performed, an SCN5A mutation was found in 27 (35.5%). Table 1 summarizes the clinical characteristics of patients included in the study.

**Table 1:** Clinical characteristics of BS patients.

	All patients (n = 105)	Symptomatic (n = 24)	Asymptomatic (n = 81)	*p*-value
**Age**, *years old*	45.17 ± 13.62	46.25 ± 15.23	44.85 ± 13.20	0.852
**Male sex**, *n (%)*	80 (76.2%)	60 (74.1%)	20 (83.3%)	0.352
**ICD implantation**, *n (%)*	42 (40%)	24 (100%)	18 (22.2%)	< 0.001
**SCN5A mutation**, *n (%)*	27 (35.5%)	6 (31.6%)	21 (36.8%)	0.680

The cardiac response to exercise is influenced by the complex interaction of many factors including age, gender, physical conditioning, sympathetic drive, baroreceptor reflexes and venous return [26]. Nevertheless, since no significant differences in age, gender and SCN5A-mutation presence between symptomatic and asymptomatic groups were noted (*p*-value>0.05), similar baseline characteristics were assumed between populations.

### Data acquisition and test

Patients underwent a triangular exercise test recommended by the American Heart Association [27] where the load was increased until it reached the 80% of the theoretical maximum heart rate of each patient, defined by the formula *MHR* = 220 − *age* [28]. The test was performed in a cyclo ergometer (Ergoline 900 Egamed, Piestany, Slovakia) and divided in the following phases:

- Exercise phase:
  - Warm-up phase: for men, initial load of 50 watts (W); for women, initial load of 30 W, both for 2 minutes.
  - Incremental exercise phase: for men, initial load of 80 W for 2 minutes and then incrementing 20 W every 2 minutes; for women, initial load of 50 W increasing load 20 W every 2 minutes.
- Recovery phase:
  - Active recovery: for men, fixed load of 50 W; for women, fixed load of 30 W, both for 3 minutes.
  - Passive recovery: total cessation of effort for 3 minutes.

The standard 12-lead ECG recordings sampled at 1000 Hz from each patient were collected and analyzed by the central board (Rennes University Hospital).

### Proposed classification methodology

**Fig 1:**
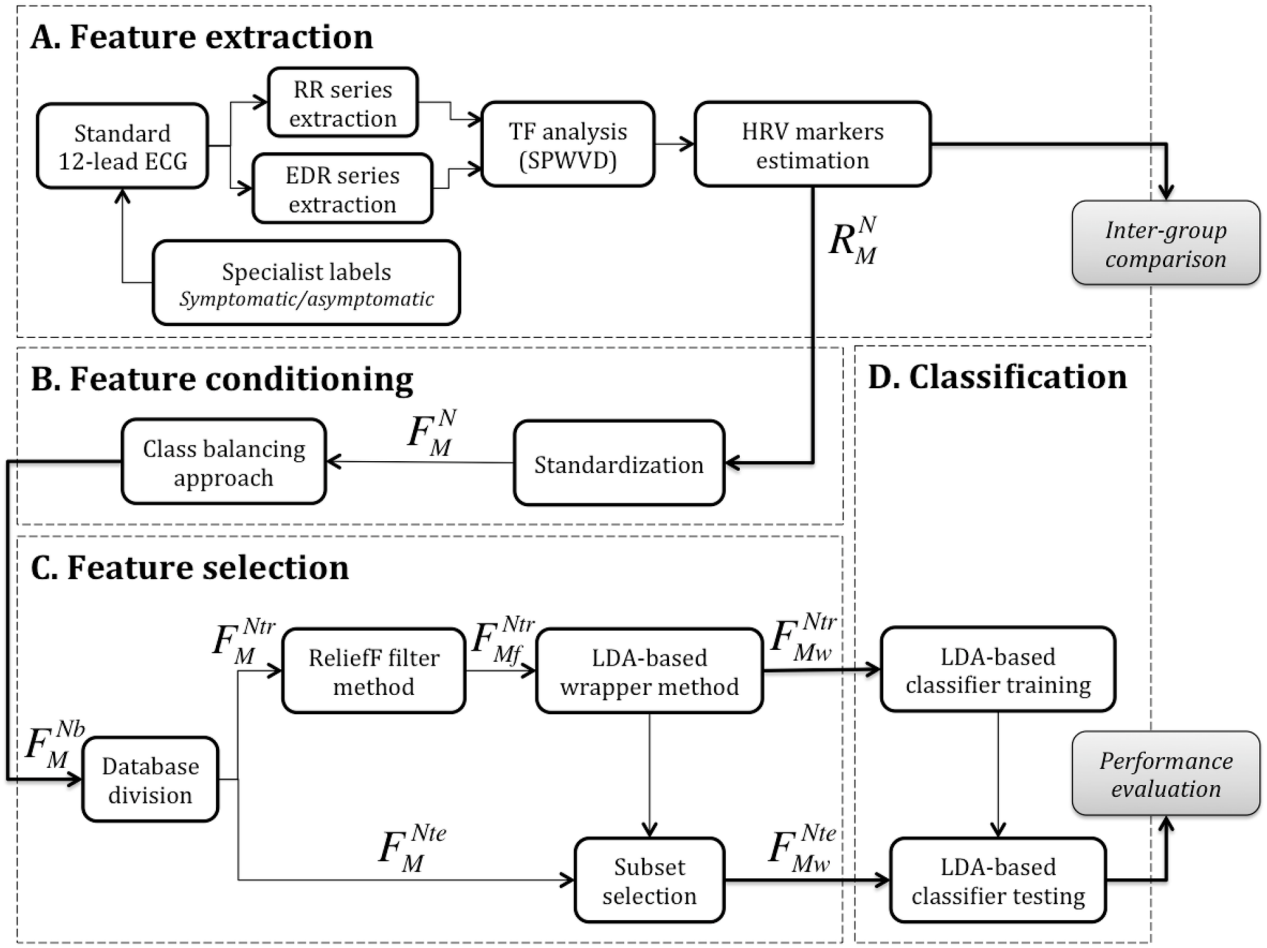
General diagram of the proposed classification methodology. Data processing is composed of four major steps. A) Feature extraction is focused on the estimation of a matrix of time-frequency HRV markers ($R_{M}^{N}$, with *N* = 105 patients and *M* = 60 different HRV features), using a time-varying frequency band that depends on the estimated instantaneous respiratory rate. B) Feature conditioning consists on standardizing and balancing $R_{M}^{N}$, leading to matrices $F_{M}^{N}$ and $F_{M}^{N_{b}}$, respectively, where *N*_*b*_ refers to the 160 observations after class balancing (79 symptomatic and 81 asymptomatic samples). C) Feature selection, which starts by randomly defining patient subsets for training, (*N*_*tr*_, 75% of patients, 59 symptomatic and 60 asymptomatic) and testing (*N*_*te*_, the rest of patients, 20 symptomatic and 21 asymptomatic), followed by the estimation of a minimal feature dimension *M*_*w*_ < *M*, that maximizes classification performance, using filtering and wrapper methods. D) The final step is dedicated to classification and performance evaluation.

Fig 1 illustrates the global methodology proposed in this paper in order to differentiate symptomatic and asymptomatic BS patients. This methodology is based on a general machine learning approach built from the following four main steps:

A Feature extraction. The standard 12-lead ECG signals acquired during exercise testing were analyzed to detect each QRS complex and extract the RR and ECG-Derived Respiration (EDR) series of each patient. A time-frequency (TF) method based on the smoothed pseudo Wigner-Ville distribution (SPWVD) of RR series that adapts frequency bands to respiratory information resulting from EDR signals was applied to estimate the evolution of different spectral HRV markers. Estimated features include the *LF*, *LF*_*nu*_, *HF*, *HF*_*nu*_ and $\frac{LF}{HF}$ mean values at different time periods of the exercise test. The output of this step is matrix $R_{M}^{N}$, which contains the calculated raw features, with *M* = 60 HRV markers for the *N* = 105 patients available on the whole database.
B Feature conditioning. In order to handle the impact of markers measured at different scales, all features of $R_{M}^{N}$ were standardized, leading to matrix $F_{M}^{N}$. Then, in order to reduce the effect of imbalanced classes, 55 synthetic symptomatic patients were generated and included in the analysis by a class balancing approach, resulting in matrix $F_{M}^{N_{b}}$, with *N*_*b*_ = 160 observations.
C Feature selection. After balancing all standardized HRV features, $F_{M}^{N_{b}}$ was divided in a training subset $F_{M}^{Ntr}$ (*Ntr* = 119, 75% randomly selected patients) and the remaining testing subset $F_{M}^{Nte}$ (*Nte* = 41). Then, a two-step feature selection process including a filter and a wrapper method was applied in order to capture the most relevant HRV features. In Fig 1C, $F_{M_{f}}^{Ntr}$ and $F_{M_{w}}^{Ntr}$ indicate the standardized training subsets kept after applying filter (*M*_*f*_ = 45) and wrapper (*M*_*w*_ = 22) methods, respectively. Based on the *M*_*w*_ selected features after training, a new standardized testing subset $F_{M_{w}}^{Nte}$ was defined.
D Classification. In the final step, a linear discriminant analysis classifier was applied to both training and testing subsets, to distinguish symptomatic vs. asymptomatic patients. After training the classifier with $F_{M_{w}}^{Ntr}$, its performance was evaluated and quantified based on $F_{M_{w}}^{Nte}$.

A more detailed description of each step is presented in the following sections.

### Feature extraction

#### RR series extraction

**Fig 2:**
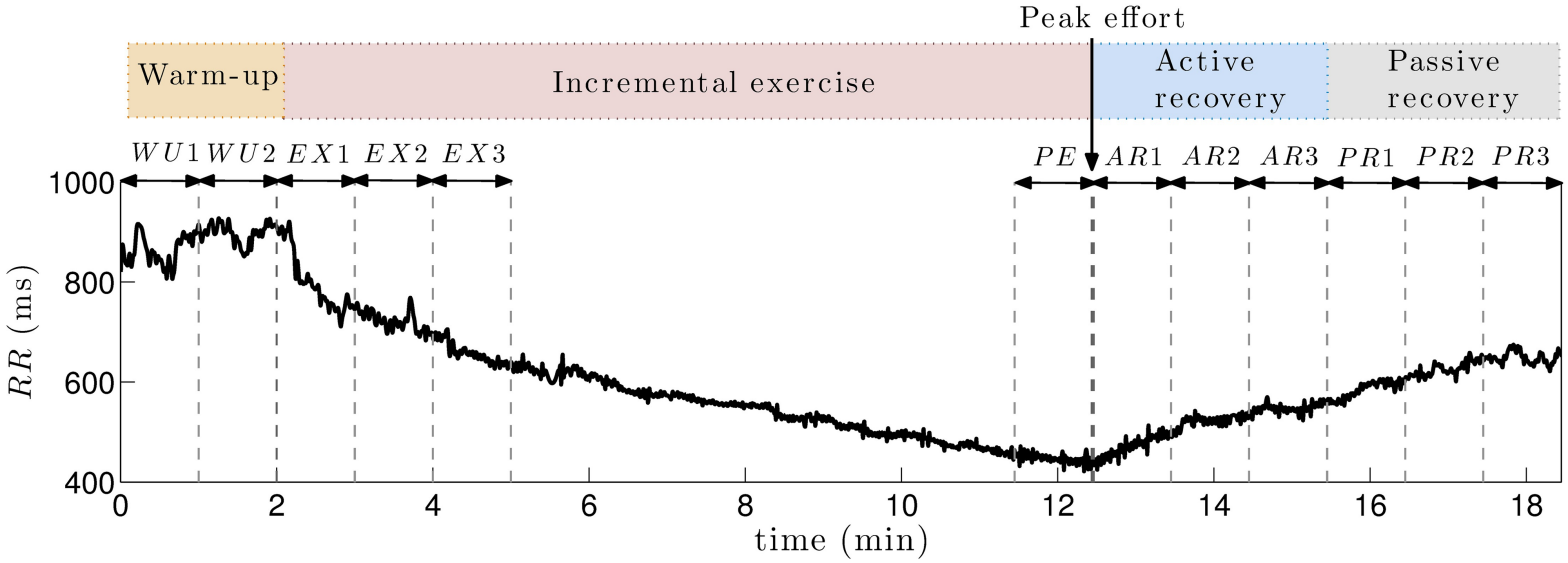
Representative example of RR series. Exercise testing was divided in four phases: warm-up, incremental exercise, active recovery and passive recovery. HRV time series estimated from the RR sequence were averaged in the following 1-minute windows: both minutes of warm-up (*WU*1 and *WU*2), first 3 minutes of incremental exercise (*EX*1, *EX*2, *EX*3), last minute of exercise before peak effort (*PE*), 3 minutes of active recovery (*AR*1, *AR*2, *AR*3) and 3 minutes of passive recovery (*PR*1, *PR*2, *PR*3).

From the standard 12-lead ECG recordings of each patient, RR-interval and R-peak amplitude series were extracted by using a noise-robust wavelet-based algorithm for QRS complex detection and subsequent R-peak location [29]. After performing manual corrections when necessary, a cubic-spline interpolation was applied to RR-interval time series, to obtain uniformly sampled data at a rate of 4 Hz. A representative example of RR series observed during each phase of the exercise test is shown in Fig 2.

#### Time-frequency HRV analysis

According to the Task Force on HRV [30], classic spectral HRV indices require stationary data to provide accurate estimates of ANS modulation. As illustrated by an increasing heart rate (decreasing RR series) in proportion to exercise workload in Fig 2, given that signals on a physical stress test are typically non-stationary, spectral characteristics associated with HRV were analyzed using a time-frequency (TF) approach.

First, in order to remove the very low frequency component, RR series were high-pass filtered at 0.03 Hz with a 4^th^ order Butterworth filter applied in both forward and backward directions so as to remove phase distortion. Then, a smoothed pseudo Wigner-Ville distribution (SPWVD) transform from the Time-Frequency toolbox [31] was employed since it has proved its usefulness for the analysis of cardiovascular signals [32].

The Wigner Ville distribution is a quadratic time-frequency method defined as the Fourier transform of the instantaneous autocorrelation function [33]. However, since it is affected by significant interference terms, the SPWVD introduces a smoothing kernel function Ψ(*τ*, *υ*), defined in Costa et al [34], that attenuates interferences while maintaining a suitable time-frequency resolution. Being *A*_*RR*_(*τ*, *υ*) the Ambiguity Function of the RR series, *x*_*RR*_(*t*), the SPWVD is defined as:

$$\begin{array}{c} A_{RR}\left(\tau ,\upsilon \right)=\int_{-\infty }^{\infty }x_{RR}(t+\frac{\tau }{2})x_{RR}^{*}(t-\frac{\tau }{2})e^{-j2\pi \upsilon t}dt \end{array}$$ (1)

$$\begin{array}{c} \psi \left(\tau ,\upsilon \right)=exp\{-\pi {\left[{\left(\frac{\upsilon }{\upsilon_{o}}\right)}^{2}+{\left(\frac{\tau }{\tau_{o}}\right)}^{2}\right]}^{2\lambda }\} \end{array}$$ (2)

$$\begin{array}{c} C_{RR}\left(t,f\right)=\int \int \psi \left(\tau ,\upsilon \right)A_{RR}\left(\tau ,\upsilon \right)e^{j2\pi (t\upsilon -\tau f)}d\upsilon d\tau \end{array}$$ (3)

Kernel parameters were adjusted to *υ*_0_ = 0.06 and *τ*_0_ = 0.03, obtaining temporal and spectral resolutions of 16.7 seconds and 0.033 Hz, respectively. Among all the analyzed combinations, this one led to the most efficient interference terms cancellation for the lowest TF filtering. Then, HRV was measured as the total power in LF and HF bands (noted as *LFb* and *HFb*), obtained from the SPWVD:

$$\begin{array}{c} LF\left(t\right)=\int_{LFb}C_{RR}\left(t,f\right)df \end{array}$$ (4)

$$\begin{array}{c} HF\left(t\right)=\int_{HFb}C_{RR}\left(t,f\right)df \end{array}$$ (5)

Assuming that sympathetic activity always lies within the standard LF band, this band was fixed between 0.04 and 0.15 Hz for the whole stress test. However, the total power in the HF band captures parasympathetic activity, closely related to respiratory sinus arrhythmia (RSA). Since respiratory frequency during exertion is not restricted to the classic HF band (0.15–0.4 Hz) and can increase up to 0.7 Hz, HRV analysis within the standard frequency band would lead to unreliable measures of the parasympathetic activity. In order to overcome this limitation, we defined a time-varying HF band, based on an estimation of the respiratory activity from the ECG signal, by applying an ECG-Derived Respiration (EDR) method [35].

#### Time-varying respiratory frequency estimation

The applied EDR method estimates respiratory information from the amplitude modulation of R-wave peaks [36]. Cubic-spline interpolation at a rate of 4 Hz was also applied to the obtained series. Then, a band-pass 4^th^ order Butterworth filter between 0.15 and 0.7 Hz was applied in both forward and backward directions to remove frequencies out of the respiratory range. The same SPWVD transform used for RR series was then applied to EDR filtered signals to estimate the instantaneous respiratory frequency.

The simplest estimation method consists in finding the frequency presenting the largest peak in the spectrum at each time instant $\hat{f}\left(t\right)$. However, in order to avoid spurious peak detections, for each time instant *t*_*k*_, the search interval was limited to frequencies between 2*δ* Hz, centered around a reference frequency *f*_*r*_(*t*_*k*_): [*f*_*r*_(*t*_*k*_) − *δ*, *f*_*r*_(*t*_*k*_) + *δ*]. This reference frequency was defined as an exponential average of previous estimates:

$$\begin{array}{c} f_{r}\left(t_{k}\right)=\beta f_{r}\left(t_{k-1}\right)+\left(1-\beta \right)\hat{f}\left(t_{k-1}\right), \end{array}$$ (6)

where *β* is the forgetting factor. As in [37], a value of *β* = 0.7 was used, based on real respiratory patterns during exercise testing and *δ* = 0.01, since respiratory frequency variations are not supposed to be faster than 0.01 Hz per 0.25 s. Moreover, to reduce the risk of spurious frequency detections in the initialization of the reference frequency, the first instantaneous respiratory frequency *f*_*r*_(*t*_0_) was selected within the standard HF band (0.15 − 0.4 Hz).

Once the estimated respiratory frequency series *f*_*r*_(*t*) was obtained, the time-varying HF band for HRV analysis was defined as *HFb*(*t*) = [*f*_*r*_(*t*) − 0.125, *f*_*r*_(*t*) + 0.125] Hz, with *t* covering the whole test.

#### Final HRV features extraction

Unlike classic autonomic indices, SPWVD leads to time-frequency HRV estimates that are indeed time series that vary during the exercise testing. These markers, accounting for the sympathetic and parasympathetic influences of the ANS on heart rate, were normalized and expressed as percentages of the total power (*TP*), defined as the sum of both spectral bands (*TP*(*t*) = *LF*(*t*) + *HF*(*t*)), leading to the time series *LF*_*nu*_(*t*) and *HF*_*nu*_(*t*):

$$\begin{array}{c} LF_{nu}\left(t\right)=\frac{LF(t)}{TP(t)}\cdot 100 \end{array}$$ (7)

$$\begin{array}{c} HF_{nu}\left(t\right)=\frac{HF(t)}{TP(t)}\cdot 100 \end{array}$$ (8)

From this definition of normalization, it should be noted that *LF*_*nu*_(*t*) = 100 − *HF*_*nu*_(*t*) and, thus, statistical results for both time series are identical. $\frac{LF}{HF}\left(t\right)$ was also calculated from dividing *LF*(*t*) by *HF*(*t*), so as to obtain the global sympathovagal balance.

Finally, all HRV estimates were averaged in temporal non-overlapped windows of 1 minute for each patient, leading to $\bar{LF^{i}}$, $\bar{LF_{nu}^{i}}$, $\bar{HF^{i}}$, $\bar{HF_{nu}^{i}}$ and $\bar{{\frac{LF}{HF}}^{i}}$, which stand for each time series intra-patient mean for the following time periods: *i* ∈ {*WU*1, *WU*2, *EX*1, *EX*2, *EX*3, *PE*, *AR*1, *AR*2, *AR*3, *PR*1, *PR*2, *PR*3}. Since each test differed in the incremental exercise phase duration and the shortest case lasted less than 5 minutes, in order to compare the same time periods between groups of patients, only the first 3 minutes of incremental exertion (*EX*1, *EX*2 and *EX*3), as well as the last minute before peak effort (*PE*), were assessed. In addition, the entire warm-up (*WU*1 and *WU*2) and both active (*AR*1, *AR*2 and *AR*3) and passive recovery (*PR*1, *PR*2 and *PR*3) phases were compared between symptomatic and asymptomatic patients. Fig 2 displays the analyzed periods along the different phases of the exercise test on a representative example of RR-interval series, indicating the peak effort instant.

From each HRV series, 12 intra-patient 1-minute means were calculated, leading to 60 features per patient that, after being compared between populations, were included in the multivariate classification approach described herein. Data in S1 Table include the resulting HRV features for the whole clinical series.

Although the autonomic response to exercise significantly depends on test conditions [38], since no statistically significant differences in workload at peak effort were observed between populations (symptomatic: 175.4 ± 56.6 W; asymptomatic: 175.1 ± 55.3 W; *p*-value: 0.957), similar exercise intensities in both groups of patients were assumed.

#### Statistical comparison

Comparisons between symptomatic and asymptomatic patients at each analyzed minute of the physical stress test were evaluated by Mann-Whitney U non-parametric tests. In order to compare the last minute of exertion and recovery, all patients had to be synchronized with respect to the peak effort instant. The analysis was made using the commercially available software MatLab (Mathworks Inc., MI, USA) and setting the level of significance at *p* < 0.05.

### Feature conditioning

All features extracted from HRV analysis were considered as candidates for the construction of a model classifying symptomatic and asymptomatic BS patients. The initial feature subset $R_{M}^{N}$ was composed of *N* = 105 observations (24 symptomatic and 81 asymptomatic patients) by *M* = 60 features (5 HRV markers for 12 analyzed minutes of test).

In order to equalize the contribution of all features to multivariate analysis, each raw HRV marker *j* for each patient *i* was standardized as follows:

$$\begin{array}{c} F_{j}^{i}=\frac{R_{j}^{i}-\mu_{j}}{\sigma_{j}}, \end{array}$$ (9)

where *μ*_*j*_ is the mean and *σ*_*j*_ the standard deviation of a specific feature *j*, taking into account the data from all patients *i* = 1, ⋯, *N*. The new standardized dataset is defined in matrix $F_{M}^{N}$.

**Fig 3:**
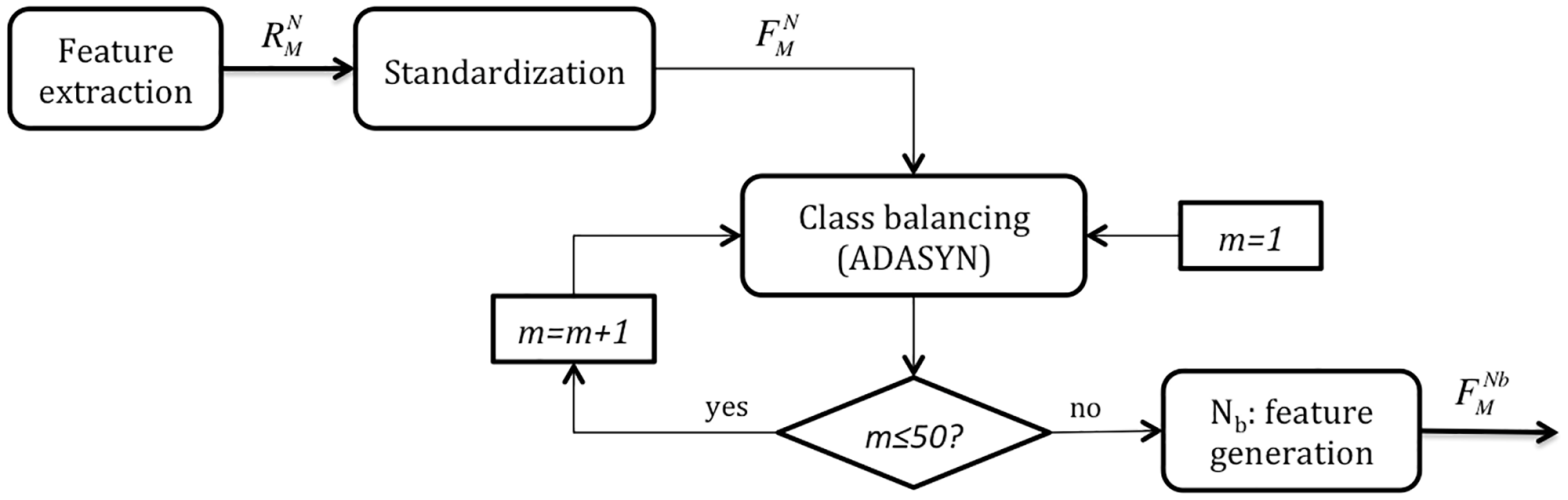
Feature conditioning. The raw feature dataset extracted from HRV analysis $R_{M}^{N}$ is standardized, leading to $F_{M}^{N}$. Then, a class balancing method is repeated 50 times, so the final value for the balanced dataset $F_{M}^{N_{b}}$ is obtained as the mean from all realizations.

Then, to attenuate the impact of imbalanced classes, synthetic symptomatic samples were generated by applying the ADASYN approach [39]. Since this method randomly chooses examples from the minority class to generate new samples, the algorithm was applied 50 times and the mean from all realizations was kept as the final balanced dataset $F_{M}^{N_{b}}$, where *N*_*b*_ = 160 observations. Fig 3 illustrates the feature conditioning process.

### Feature selection

To reduce the number of attributes included in the model so as to decrease its computational cost, we applied a two-step feature selection approach that identified the most relevant features in distinguishing between symptomatic and asymptomatic patients. As previously mentioned, Fig 1 specifies the methodology followed for feature extraction and posterior feature selection, only applied to a randomly selected sample of 75% of the feature database ($F_{M}^{Ntr}$: training subset). The remaining 25% ($F_{M}^{Nte}$: testing subset) was then used for model validation.

#### ReliefF filter method

The first step in the feature selection process was a simple filter method based on the ReliefF algorithm [40]. Since this approach ignores the effects of attributes on classification, it can rapidly remove some irrelevant and redundant features.

The algorithm estimates feature weights *W* according to their capability of distinguishing between data from different classes, here symptomatic and asymptomatic patients, based on a k-Nearest Neighbors (k-NN) approach. The extension of the original Relief method used in this study, ReliefF, not only deals with multiclass problems but it is also more robust with incomplete and noisy data.

Hence, the algorithm assigns a relevance weight ranged from −1 to 1 to each feature, with large positive weights allocated to significant attributes. However, it should be noted that, since this method is based on a k-NN approach, feature weights usually depend on *k*. For small values of *k*, the estimates can be unreliable for noisy data; while for *k* values comparable with the number of observations, the algorithm can fail to find significant attributes. Thus, ReliefF was computed for *k* = 10, ⋯, 19 and *W* was defined as the average of all weights.

**Fig 4:**
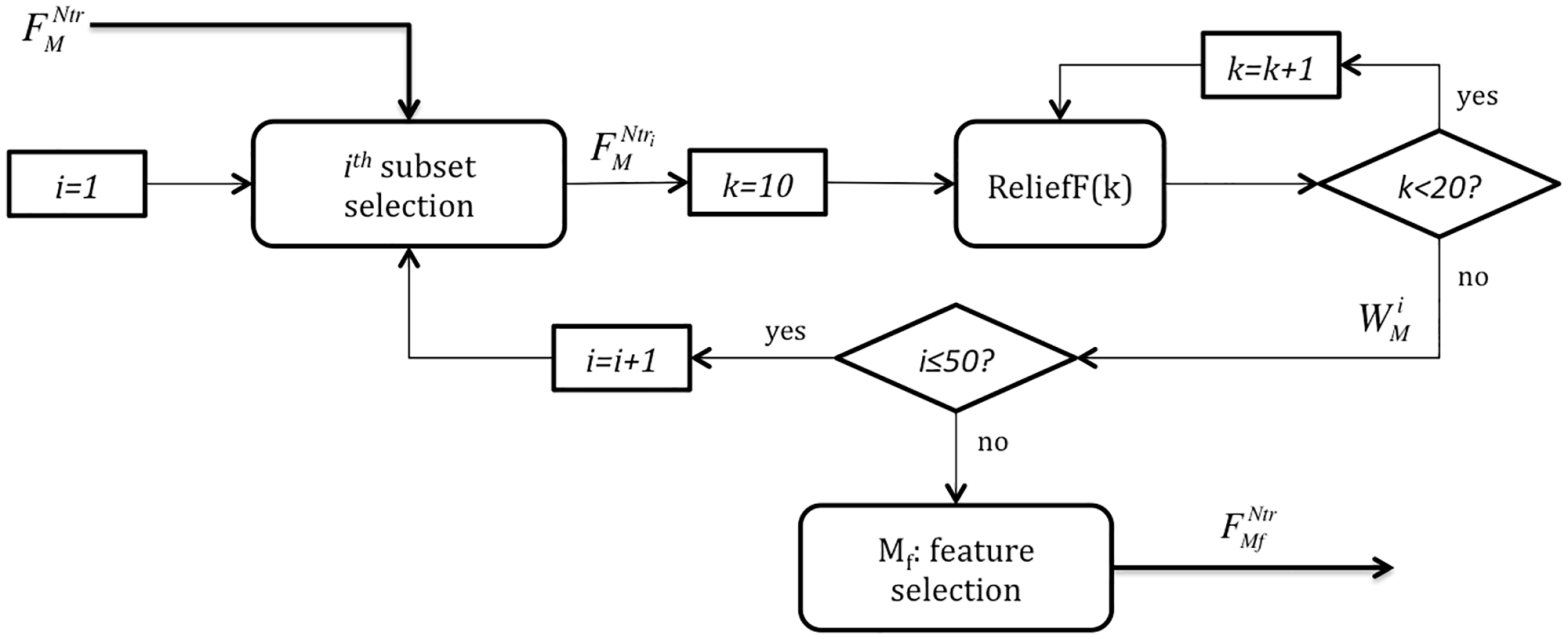
Filter feature selection based on the ReliefF algorithm. In this step, the complete training dataset $F_{M}^{Ntr}$ is employed. For each iteration *i*, a random subset composed of 60% of the training data (35 symptomatic and 36 asymptomatic observations) is defined. The weight of all features $W_{M}^{i}$ in this subset is computed as the averaged weight along *k* = 10, ⋯, 19. This process is repeated 50 times and the final weight of each feature is obtained as the median from all realizations. Finally, the *M*_*f*_ = 45 most relevant features are selected and $F_{M_{f}}^{Ntr}$ is kept as input for the wrapper feature selection step.

Moreover, since HRV feature values significantly vary over patients, a bootstrap technique was applied [41]. The algorithm was run 50 times on different randomly chosen subsets including 60% of the training dataset $F_{M}^{Ntr}$ (35 symptomatic and 36 asymptomatic observations), here represented as $F_{M}^{Ntr_{i}}$. Then, the relevance of each feature was obtained as the median of the 50 realizations analyzed. Fig 4 displays the methodology followed to select *M*_*f*_ as the 75% most relevant attributes (45 best ranked out of 60 features) that were kept for further analysis.

#### LDA-based wrapper method

For final feature selection, a second step was applied on the reduced subset of attributes resulting from the previous stage. It consisted in a wrapper algorithm with both forward and backward search strategies (floating method), using a Linear Discriminant Analysis (LDA) classifier as a black box. Since this approach is based on classification performance, the final subset is only optimized for this particular classifier [42].

**Fig 5:**
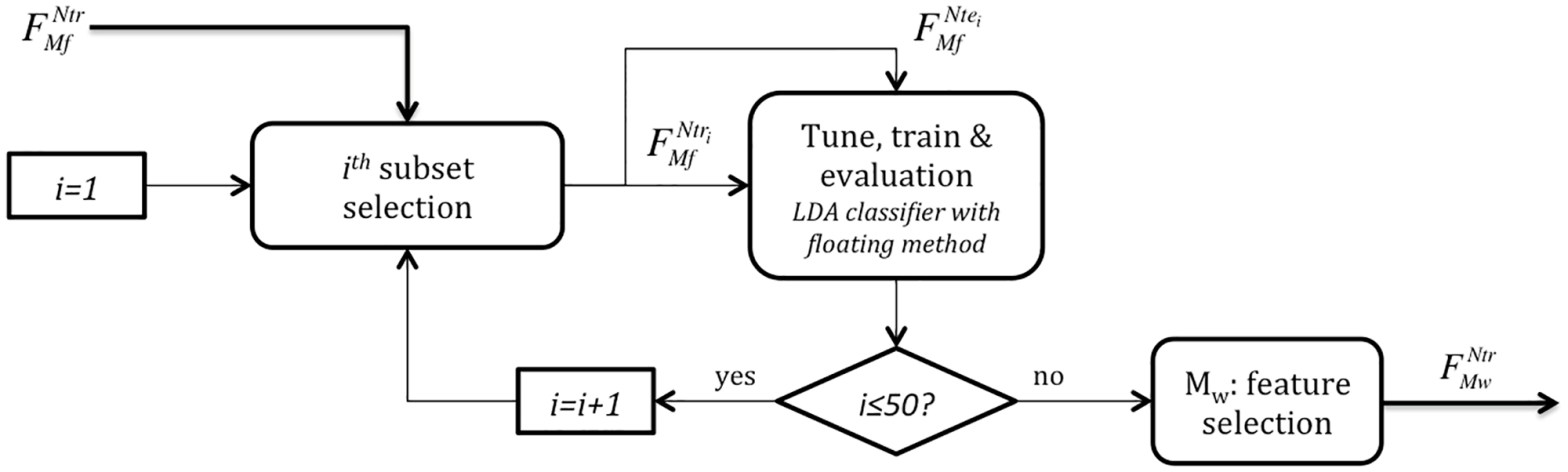
Wrapper feature selection based on the floating method and an LDA classifier. Starting from the training set obtained from the filter feature selection step $F_{M_{f}}^{Ntr}$, for each iteration *i*, a subset with 75% of the data $F_{M_{f}}^{Ntr_{i}}$ is chosen (45 symptomatic and asymptomatic samples) to tune and train an LDA-based classifier, then evaluated with the remaining 25% of the random subset $F_{M_{f}}^{Nte_{i}}$ (15 symptomatic and asymptomatic). The strategy is repeated 50 times and those attributes appearing more than *L* times among all realizations are kept for the final feature subset $F_{M_{w}}^{Ntr}$.

Fig 5 represents the wrapper feature selection process in more detail. As in the previous step, it was repeated 50 times on different randomly chosen subsets of training data $F_{M_{f}}^{Ntr_{i}}$. Those features appearing more than *L* times, among the 50 realizations, formed the final subset $F_{M_{w}}^{Ntr}$. The value of *L* was optimized, based on performance metrics, so as to find the best selection of features *M*_*w*_ leading to the finest classifier distinguishing between symptomatic and asymptomatic BS patients.

### LDA-based classifier

After selecting the best set of features, an LDA classifier [43] was implemented using a 5-fold cross-validation approach in order to reduce classification error. This technique divides the entire training subset $F_{M_{w}}^{Ntr}$ into 5 blocks where each classifier is firstly trained on 4 portions and then tested on the 5*^th^* block. This is performed for the 5 different possible combinations of blocks for training/testing so the outputs of each solution are then averaged.

**Fig 6:**
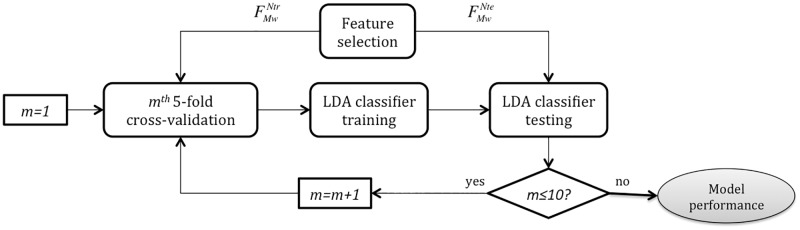
Scheme for LDA classifier training and testing, based on a 5-fold cross-validation strategy. After feature extraction, conditioning and selection, a 5-fold cross-validation was run 10 times in order to assess the classifier performance, tuned using the training subset $F_{M_{w}}^{Ntr}$ and evaluated on the testing subset $F_{M_{w}}^{Nte}$.

In addition, to estimate the mean performance variability of the classifier when applied to testing data, 5-fold cross-validation was run 10 times on differently divided subsets of training data. Fig 6 illustrates the steps followed to train and test the LDA classifier. As specified in previous sections, 75% of the data $F_{M_{w}}^{Ntr}$ were used for training, and the remaining 25% $F_{M_{w}}^{Nte}$ for testing.

### Performance evaluation

Model performance evaluation was based on the resulting confusion matrix, which specifies the number of true positives (*TP*), true negatives (*TN*), false positives (*FP*) and false negatives (*FN*), when comparing true and predicted labels and considering symptomatic patients as positives.

First, the AUC or area under the ROC (Receiver Operating Characteristic) curve was computed to quantify the classifier performance. This measure was also assessed in order to optimize the threshold *L* that led to the best performing classifier after wrapper feature selection.

Moreover, classical sensitivity (*Se* = *TP*/(*TP* + *FN*)) and specificity (*Sp* = *TN*/(*TN* + *FP*)) measures, associated with the optimal operating point in the ROC curve, were calculated to quantify the classifier capability of correctly detecting symptomatic and asymptomatic patients, respectively.

## Results

### Single-patient representation

Given that all patients presented similar tendencies in RR and HRV series, the following representative example illustrates changes induced by exercise testing on those time series involved in HRV features extraction.

**Fig 7:**
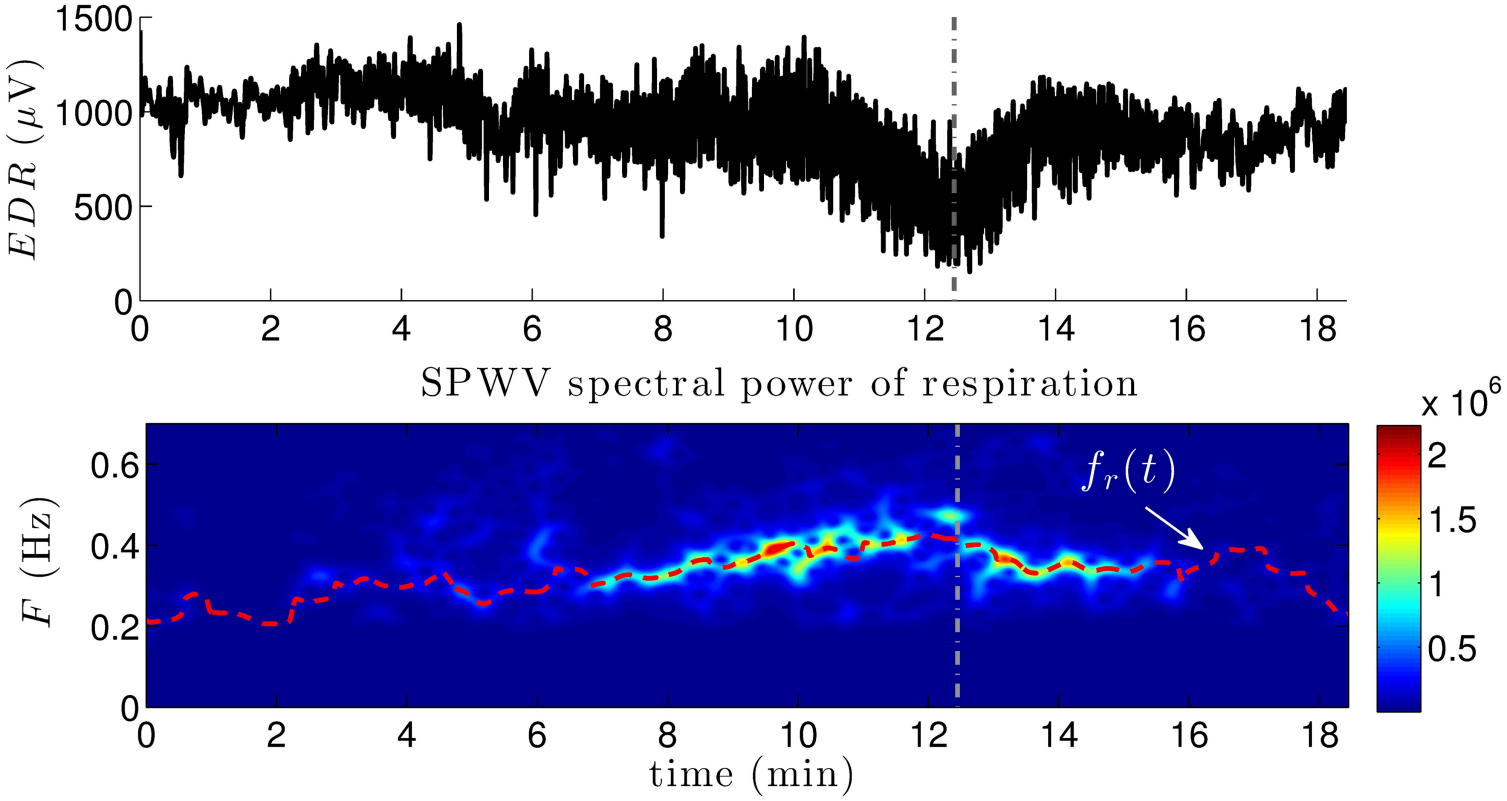
Representative example of respiration information. The upper panel represents an example of EDR series, calculated from the R-wave peak amplitudes of a specific patient. The SPWVD spectral power associated is displayed in the second panel, along with the instantaneous respiratory frequency *f*_*r*_(*t*) estimated from a corrected version of the frequencies presenting the maximum spectral powers at each time instant (dashed red line). Vertical dashed lines indicate peak effort before recovery.

The upper panel of Fig 7 displays an example of EDR series. Below, the SPWVD spectral power of respiration is shown, along with its estimated instantaneous respiratory frequency *f*_*r*_(*t*), represented with a dashed red line. Note that, in this example, as the patient approaches the peak effort (dashed vertical line), respiratory frequency exceeds the standard HF band upper limit of 0.4 Hz.

**Fig 8:**
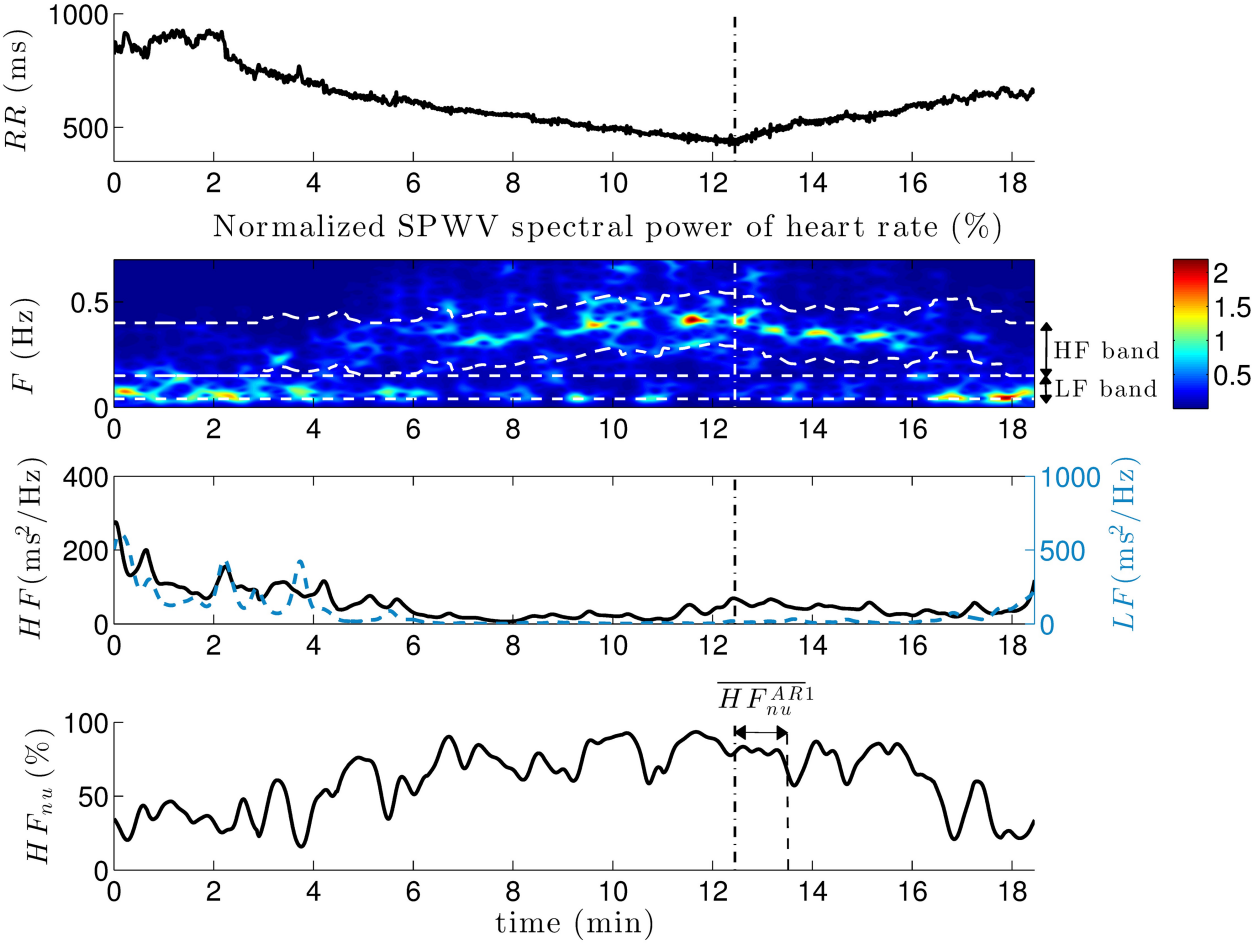
Representative example of heart rate information. From the RR series, the normalized SPWVD spectral power is calculated. Dashed white lines in the second panel indicate LF and HF bands. In the third panel, the total power in LF (blue dashed line) and HF (black solid line) bands at each time instant are represented. Then, an example of normalized time series (*HF*_*nu*_) accounts for the parasympathetic contribution along the exercise test. A vertical dashed line in all panels refers to the peak effort instant. In the last panel, the time period used to calculate $\bar{HF_{nu}^{AR1}}$ is indicated.

Based on this respiratory information, the time-varying patient-specific HF band was identified. Fig 8 displays the RR series and its associated SPWVD spectral power for the same patient, where the LF and respiration-centered HF bands are represented in dashed white lines (second panel).

Although certain stationarity can be observed during the first two minutes corresponding to the warm-up phase, the RR series displays a significant non-stationarity represented by a progressive decrease during incremental exercise and a continuous increase at recovery. As previously reported by [44], the cardiovascular response properly balanced the performed physical activity intensity. Moreover, it should be noted the non-negligible power centered in the HF band as a result of respiration. Since HF exceeds the standard band, the use of classic spectral limits would have led to unreliable measures of the parasympathetic activity in this patient.

The third panel shows the time series *LF*(*t*) and *HF*(*t*) extracted from TF analysis, then normalized and expressed as percentages of the total power. Finally, the last panel displays the obtained *HF*_*nu*_(*t*) series, where the 1-minute window to calculate the mean value of *HF*_*nu*_ during the first minute of active recovery ($\bar{HF_{nu}^{AR1}}$) is indicated. The results show a progressive increase in *HF*_*nu*_(*t*) during exercise, as well as a decrease at recovery. Although not represented in Fig 8, due to the normalization step applied to *LF*(*t*) and *HF*(*t*), the complementary effect was observed for *LF*_*nu*_(*t*).

### Inter-group comparison

HRV features extracted during exercise and recovery were compared between symptomatic and asymptomatic patients. Apart from warm-up and recovery phases, since exercise duration was highly variable among subjects, only the first 3 minutes and the last minute of incremental exertion were analyzed.

During the second minute of incremental exercise, statistically significant differences were found in mean normalized HF ($\bar{HF_{nu}^{EX2}}$, *p* = 0.041) and thus in $\bar{LF_{nu}^{EX2}}$. Symptomatic patients showed an increased $\bar{HF_{nu}}$, and a reduced $\bar{LF_{nu}}$, in this time period with respect to asymptomatic patients. However, no significant differences between groups were noted after exertion, during active or passive recovery.

Table 2 summarizes the mean, standard deviation and associated *p*-values for $\bar{LF}$, $\bar{LF_{nu}}$, $\bar{HF}$, $\bar{HF_{nu}}$ and $\bar{\frac{LF}{HF}}$ obtained during the warm-up phase, the first 3 minutes of incremental exercise and the last minute before peak effort.

**Table 2:** Mean ± standard deviation, for symptomatic and asymptomatic patients, and associated *p*-values of HRV markers during exercise.

	Warm-up		Incremental exercise			
	Minute 1	Minute 2	Minute 1	Minute 2	Minute 3	Last minute
$\bar{LF}$ [ms^2^/Hz]						
*Symptomatic*	2245.6±4063.2	1749.2±2321.6	738.95±801.84	228.93±302.24	129.69±199.10	10.39±28.69
*Asymptomatic*	1392.7±1605.4	1202.4±1330.0	635.19±685.42	442.88±772.15	250.03±509.18	35.90±205.70
*p* − *value*	0.746	0.298	0.985	0.161	0.113	0.894
$\bar{LF_{nu}}$ [%]						
*Symptomatic*	75.08±18.08	76.21±16.97	70.48±17.58	65.82±17.97	65.46±17.98	49.68±21.09
*Asymptomatic*	77.98±11.56	77.95±11.33	78.09±11.30	74.45±12.56	71.55±13.86	52.04±20.67
*p* − *value*	0.918	0.852	0.062	0.041*	0.173	0.728
$\bar{HF}$ [ms^2^/Hz]						
*Symptomatic*	1343.1±4819.3	421.62±779.60	227.66±491.88	209.78±738.12	144.06±509.70	41.44±175.75
*Asymptomatic*	399.37±842.57	309.40±401.34	180.57±366.29	133.22±292.49	71.84±124.47	11.23±44.85
*p* − *value*	0.499	0.519	0.221	0.804	0.728	0.781
$\bar{HF_{nu}}$ [%]						
*Symptomatic*	24.92±18.94	23.80±16.97	29.52±17.58	34.18±17.97	34.54±17.98	50.32±21.09
*Asymptomatic*	22.02±11.56	22.05±11.33	21.91±11.30	25.55±12.56	28.45±13.86	47.96±20.67
*p* − *value*	0.918	0.852	0.062	0.041*	0.173	0.728
$\bar{LF/HF}$ [unitless]						
*Symptomatic*	7.96±8.11	8.78±9.38	6.87±9.43	3.75±3.61	3.09±2.33	1.71±1.73
*Asymptomatic*	5.89±4.11	5.90±5.72	6.00±5.29	4.90±3.91	4.11±2.87	4.32±21.53
*p* − *value*	0.816	0.763	0.157	0.053	0.122	0.661

*p < 0.05, Mann-Whitney U test.

### Classification

**Fig 9:**
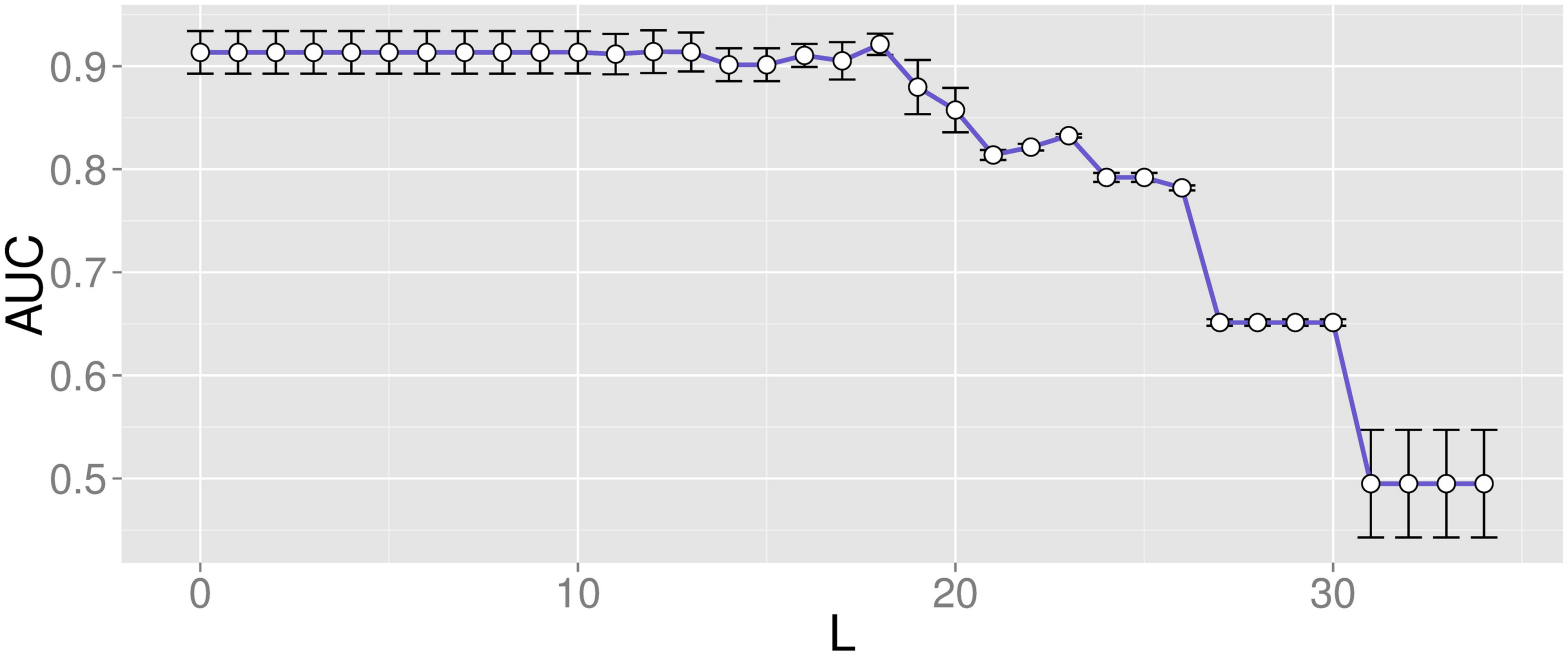
Mean and standard deviation of *AUC* for each value of *L*. Performance evaluation with features appearing more than *L* times.

After feature conditioning, feature selection was performed by a two-step approach. Filter selection was followed by the repeated application of a wrapper method to the selected features. The final subset contained those features appearing more than a specific number of times *L* among realizations, optimized based on the performance metric *AUC*. Fig 9 displays the mean and standard deviation of the *AUC* associated with each value of *L*.

Based on these results, the final classifier placed the threshold at *L* = 18, where a maximum *AUC* using the minimum number of features was found. When only those parameters appearing more than 18 times were kept, the final subset contained 22 features (listed in Table 3), leading to an *AUC* = 0.92 ± 0.01, *Se* = 0.91 ± 0.06 and *Sp* = 0.90 ± 0.05.

**Table 3:** Selected features from wrapper method.

1	2	3	4	5	6	7	8	9	10	11
$\bar{HF_{nu}^{WU1}}$	$\bar{HF_{nu}^{EX3}}$	$\bar{HF_{nu}^{PE}}$	$\bar{HF_{nu}^{AR1}}$	$\bar{HF_{nu}^{AR3}}$	$\bar{HF_{nu}^{PR1}}$	$\bar{{\frac{LF}{HF}}^{AR2}}$	$\bar{LF^{EX1}}$	$\bar{LF^{EX2}}$	$\bar{LF^{EX3}}$	$\bar{LF^{AR3}}$
12	13	14	15	16	17	18	19	20	21	22
$\bar{LF^{PR2}}$	$\bar{LF^{PR3}}$	$\bar{HF^{PE}}$	$\bar{HF^{AR3}}$	$\bar{HF^{PR2}}$	$\bar{HF^{PR3}}$	$\bar{LF_{nu}^{EX3}}$	$\bar{LF_{nu}^{PE}}$	$\bar{LF_{nu}^{AR1}}$	$\bar{LF_{nu}^{AR2}}$	$\bar{LF_{nu}^{AR3}}$

**Fig 10:**
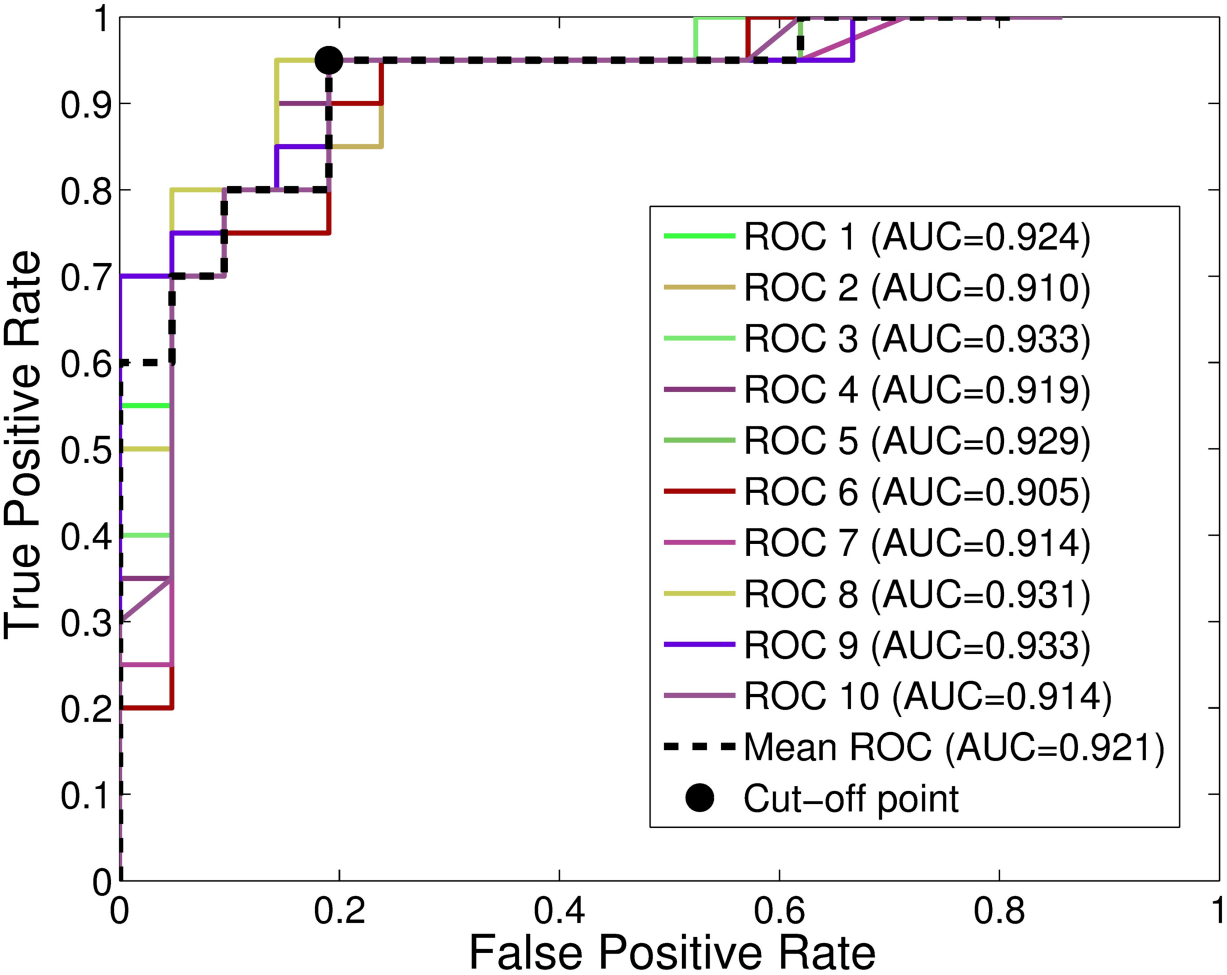
ROC curves and associated *AUC* values for *L* = 18. Mean ROC curves for each cross-validation and global ROC, when features appearing more than 18 times are kept.

Fig 10 displays the mean ROC curves resulting from each 5-fold cross-validation, as well as the global ROC curve and its optimal operating point, for the proposed classifier.

## Discussion

In this study, the autonomic response to exercise testing was analyzed in 105 BS patients. Although many previous studies have already assessed the autonomic function in BS, only a few have exploited the potential of exertion to predict cardiac events [22–25]. Moreover, since symptoms have been related to VF occurrence on this population [5, 6], our aim was to compare, according to symptomatic status, the time-varying HRV changes induced by effort and subsequent recovery. To our knowledge, this work presents the first time-frequency HRV analysis under conditions of physical activity in BS. Furthermore, due to the complexity of distinguishing between symptomatic and asymptomatic patients by means of univariate analyses, a multivariate classifier based on the combination of the extracted HRV features was proposed.

Most reported approaches assessing the autonomic response of BS patients are based on measurements obtained from 24-hour recordings that lead to controversial conclusions. In a clinical series of 17 patients with BS, of whom 10 were asymptomatic subjects with Brugada ECG, and 45 controls, Krittayaphong et al [12] concluded that BS patients presented a lower HRV and a lower vagal tone at night compared to controls, as well as lower diurnal and higher overnight heart rates compared to asymptomatic subjects and controls. Likewise, Hermida et al [13] reported a significantly lower HRV at night in 21 symptomatic, with respect to 26 asymptomatic BS patients. Pierre et al [14] also asserted in a clinical series of 46 BS patients and 46 controls that HRV in the first group was significantly lower with respect to healthy subjects. Tokuyama et al [15] results showed a significantly lower HRV in BS patients when analyzing a series of 12 symptomatic, 17 asymptomatic and 16 healthy individuals. The results also reflected a significant reduction in both sympathetic and parasympathetic tones in BS patients, as well as a decreased circadian variation of the autonomic function over 24 hours, with respect to controls. Kostopoulou et al [17] examined autonomic disorders in 20 patients with BS and 20 age-matched controls. In that case, HRV analysis did not reveal any significant difference between groups, but a high susceptibility to vasovagal syncope was observed in BS patients, possibly being a disease-related symptom. Nakazawa et al [18] analyzed, using a 24-hour continuous ECG monitoring, the autonomic properties of 27 BS patients (10 of them had a history of VF and 17 did not) and of 26 healthy subjects, finding higher vagal and reduced sympathetic tones in symptomatic BS patients. Likewise, in a recent work from our group where the 24-hour Holter recordings from 118 BS patients were analyzed, symptomatic subjects showed an increased parasympathetic activity during both daytime and nighttime [16]

Subramanian et al [23] proved the usefulness of some electrocardiographic markers extracted during exercise testing for risk stratification in BS. Moreover, our group has recently reported significant differences in heart rate complexity between symptomatic and asymptomatic patients, during periods of recovery after exertion [24]. Nevertheless, Amin et al [22] published the first work measuring the autonomic function of 50 BS patients and 35 controls during exercise, finding a higher parasympathetic reactivation during early recovery in patients with prior VF events. Likewise, Makimoto et al [21] analyzed the autonomic function of 93 BS patients and 102 controls during recovery from treadmill exercise testing. They studied parasympathetic reactivation by computing the Heart Rate Recovery (HRR) after peak exercise, concluding that a higher vagal activity was related to the occurrence of cardiac events in BS.

In our study, as illustrated in Fig 8, all patients displayed a progressive increase in the mean normalized HF ($\bar{HF_{nu}}$) during incremental exercise, as well as an $\bar{HF_{nu}}$ decrease at recovery. Although data on direct sympathetic nerve recording and plasma catecholamines measurements have reported that a decreased parasympathetic and an increased sympathetic activity play a major role in the autonomic response to exercise, many studies on cardiac autonomic function based on HRV analysis have failed to represent this response, even in healthy subjects [38]. Indeed, the *LF* component does not provide an index of sympathetic tone but rather reflects a complex interplay among many factors including the sympathetic and parasympathetic contributions to ANS. Similarly, just as parasympathetic neural activity influences *LF* values, sympathetic activation also modulates the *HF* component [45]. Moreover, since an increasing power can be noted at the HF band center corresponding to the respiratory frequency (second panel in Fig 8), the gradual increase observed in $\bar{HF_{nu}}$ during incremental exercise may be significantly influenced by respiration. Thus, *LF* and *HF* indices should not be analyzed as accurate representations of, respectively, the sympathetic and parasympathetic tones. They should be interpreted as estimates of the autonomic function that may capture relevant tendencies in HR modulation, potentially useful for the detection of differences between BS patients at different levels of risk.

According to the inter-group comparison of HRV markers, statistically significant differences were observed during the second minute of incremental exercise in $\bar{HF_{nu}}$, and thus $\bar{LF_{nu}}$, between groups. Since no significant differences were found in terms of spectral power at the respiratory frequency, *HF*_*nu*_ differences between populations might mostly be due to vagal activity. Thus, as previously reported [15, 16, 18], symptomatic patients seem to experience an increased parasympathetic modulation with respect to asymptomatic patients, supporting the idea that higher vagal responses could be related to a worse prognosis in BS. These results may be explained by the dysfunction on presynaptic norepinephrine (NE) recycling and the reduction in the concentration of NE at the synaptic cleft found on previous works based on positron emission tomography on BS patients [8–11].

The lack of significant results in univariate analysis reveals the difficulty of distinguishing between symptomatic and asymptomatic groups. Therefore, a multivariate approach following a step-based machine learning method was designed in order to improve classification performance. The proposed solution significantly reduced the final subset of features included in the predictive LDA-based model to one third of the total amount of HRV features, leading to a mean *AUC* of 92.1%. First, a filter feature selection method discarded the least relevant and most redundant features, holding the 75% of the initial features subset, to which the LDA-based wrapper algorithm was applied. On the one hand, the results after filtering show that all autonomic markers during the last minutes of incremental exercise and recovery were kept, evidencing the relevance of these test segments in classifying BS patients. On the other hand, although $\bar{HF_{nu}^{EX2}}$ led to significant results in univariate analysis, the applied filter method identified this marker as a redundant feature and only kept $\bar{LF_{nu}^{EX2}}$ for further analysis.

Since classification performance significantly depends on the number of chosen features after wrapper feature selection, the algorithm was optimized to obtain the best *AUC* using the minimum number of features. Thus, when selecting only those features appearing more than 18 times after wrapper application, an optimal classifier containing 22 parameters was implemented. Among the final subset of features, only one $\frac{LF}{HF}$ marker was kept, acquired during the second minute of active recovery. The remaining parameters equally belonged to *LF* and *LF*_*nu*_ or *HF* and *HF*_*nu*_ measures. Regarding test phases, only one feature came from the warm-up phase ($\bar{HF_{nu}^{WU1}}$) and other 5 markers were acquired during the passive recovery stage. Most parameters were measured during incremental exercise and active recovery, and more specifically during the last minutes of both phases.

Recent studies have also proposed prediction models for VA risk stratification in BS patients using non-invasive parameters [46]. However, our model outperformed previous approaches, evidencing the interest of analyzing HRV features during exercise testing to better understand VF risk in this population. Indeed, the results from our previous study, where the classification potential of these markers was already presented [47], were also improved by enlarging the clinical series under study and applying a more robust feature selection approach.

This study presents some relevant limitations that should be noted. The clinical value of autonomic features can only be proved if a close relationship between HRV markers and ventricular events is established. Since no VF was induced during the test, HRV variations in symptomatic patients cannot be directly related to this phenomenon. Moreover, a synthetic oversampling approach was applied in order to overcome complications found when learning from imbalanced datasets. Thus, the obtained results should be validated by enlarging the studied population. Finally, since some asymptomatic patients may develop symptoms in the future and thus present high-risk patterns during the analyzed recordings, a more suitable clinical database for risk stratification should include follow-up information. Thereby, autonomic changes could have been related to the probability of developing symptoms rather than to the identification of a high-risk group having already suffered these symptoms.

Nonetheless, previous studies have shown the need of new autonomic markers with higher predictive values, such as those here presented, to better stratify risk in patients suffering from Brugada syndrome. According to international guidelines [1], ICD implantation is recommended in BS patients being survivors of a cardiac arrest and/or having documented spontaneous sustained ventricular tachycardias (class I) and can be useful in patients with a spontaneous diagnostic type 1 Brugada-like ECG pattern having a history of syncope caused by ventricular arrhythmias (class IIa). However, the decision of implanting an ICD on asymptomatic subjects is still contentious, even if they represent around the 60% of diagnosed patients. Thus, the proposed model is presented as a potential instrument to better identify those asymptomatic BS patients at high risk who may benefit from an ICD implantation. Moreover, the proposed model might also be used for processing HRV data acquired from ICDs on implanted BS patients, in order to control their risk of VF occurrence during follow-up.

## Conclusions

In this study, the autonomic function of 105 BS patients who underwent a standardized physical stress test was analyzed so as to characterize symptomatic and asymptomatic populations. Based on the hypothesis that changes in the ANS induced by exercise testing could improve prognosis interpretation, a classifier capable of identifying patients at high risk was then designed.

First, the extracted time-varying HRV features were compared between populations. Statistically significant differences were found in $\bar{LF_{nu}}$ and $\bar{HF_{nu}}$ during incremental exercise, suggesting that symptomatic patients seem to experience an increased vagal function with respect to asymptomatic BS patients.

Then, a predictive model based on a two-step feature selection strategy identified the most discriminant HRV features to distinguish symptomatic and asymptomatic patients. Despite the difficulty in finding differences between these populations, classification results show the potential of autonomic markers when identifying symptoms in BS.

Although the present study presents some limitations and is based on a relatively small population of BS patients, the results indicate important trends of clinical relevance that could be useful for risk stratification in asymptomatic patients for whom the decision to implant a cardioverter defibrillator is complex and controversial.

## Supporting information

S1 TableHRV markers resulting from feature extraction.Estimated features include the *LF*, *LF*_*nu*_, *HF*, *HF*_*nu*_ and $\frac{LF}{HF}$ mean values at different time periods of the exercise test, leading to 60 HRV features for the 105 BS patients under study.(XLSX)

## References

[1] Priori S, Blomström-Lundqvist C, Mazzanti A, Blom N, Borggrefe M, Camm J, et al Task Force for the Management of Patients with Ventricular Arrhythmias and the Prevention of Sudden Cardiac Death of the European Society of Cardiology (ESC). 2015 ESC guidelines for the management of patients with ventricular arrhythmias and the prevention of sudden cardiac death: the Task Force for the Management of Patients with Ventricular Arrhythmias and the Prevention of Sudden Cardiac Death of the European Society of Cardiology (ESC) endorsed by: Association for European Paediatric and Congenital Cardiology (AEPC). Europace. 2015;17:1601–1687. doi: 10.1093/europace/euv319 26318695 10.1093/europace/euv319

[2] Priori SG, Wilde AA, Horie M, Cho Y, Behr ER, Berul C, et al Executive summary: HRS/EHRA/APHRS expert consensus statement on the diagnosis and management of patients with inherited primary arrhythmia syndromes. Europace. 2013;15(10):1389–1406. doi: 10.1093/europace/eut272 23994779 10.1093/europace/eut272

[3] Brugada P, Brugada J. Right bundle branch block, persistent ST segment elevation and sudden cardiac death: a distinct clinical and electrocardiographic syndrome: a multicenter report. Journal of the American College of Cardiology. 1992;20(6):1391–1396. doi: 10.1016/0735-1097(92)90253-J 1309182 10.1016/0735-1097(92)90253-j

[4] Brugada J, Brugada R, Brugada P. Right bundle-branch block and st-segment elevation in leads V1 through V3 a marker for sudden death in patients without demonstrable structural heart disease. Circulation. 1998;97(5):457–460. doi: 10.1161/01.CIR.97.5.457 9490240 10.1161/01.cir.97.5.457

[5] Antzelevitch C. Heart Rhythm Society; European Heart Rhythm Association. Brugada syndrome: report of the second consensus conference: endorsed by the Heart Rhythm Society and the European Heart Rhythm Association. Circulation. 2005;111(5):659–670. doi: 10.1161/01.CIR.0000152479.54298.51 15655131 10.1161/01.CIR.0000152479.54298.51

[6] Probst V, Veltmann C, Eckardt L, Meregalli P, Gaita F, Tan H, et al Long-term prognosis of patients diagnosed with Brugada syndrome results from the FINGER Brugada Syndrome Registry. Circulation. 2010;121(5):635–643. doi: 10.1161/CIRCULATIONAHA.109.887026 20100972 10.1161/CIRCULATIONAHA.109.887026

[7] Matsuo K, Kurita T, Inagaki M, Kakishita M, Aihara N, Shimizu W, et al The circadian pattern of the development of ventricular fibrillation in patients with Brugada syndrome. European heart journal. 1999;20(6):465–470. doi: 10.1053/euhj.1998.1332 10213350 10.1053/euhj.1998.1332

[8] Kies P, Wichter T, Schäfers M, Paul M, Schäfers KP, Eckardt L, et al Abnormal myocardial presynaptic norepinephrine recycling in patients with Brugada syndrome. Circulation. 2004;110(19):3017–3022. doi: 10.1161/01.CIR.0000146920.35020.44 15520312 10.1161/01.CIR.0000146920.35020.44

[9] Wichter T, Matheja P, Eckardt L, Kies P, Schäfers K, Schulze-Bahr E, et al Cardiac autonomic dysfunction in Brugada syndrome. Circulation. 2002;105(6):702–706. doi: 10.1161/hc0602.103677 11839625 10.1161/hc0602.103677

[10] Paul M, Meyborg M, Boknik P, Gergs U, Schmitz W, Breithardt G, et al Autonomic dysfunction in patients with Brugada syndrome: further biochemical evidence of altered signaling pathways. Pacing and Clinical Electrophysiology. 2011;34(9):1147–1153. doi: 10.1111/j.1540-8159.2011.03127.x 21605134 10.1111/j.1540-8159.2011.03127.x

[11] Bigi MAB, Aslani A, Aslani A. Significance of cardiac autonomic neuropathy in risk stratification of Brugada syndrome. Europace. 2008;10(7):821–824. doi: 10.1093/europace/eum272 18089621 10.1093/europace/eum272

[12] Krittayaphong R, Veerakul G, Nademanee K, Kangkagate C. Heart rate variability in patients with Brugada syndrome in Thailand. European Heart Journal. 2003;24(19):1771–1778. doi: 10.1016/j.ehj.2003.06.005 14522573 10.1016/j.ehj.2003.06.005

[13] Hermida JS, Leenhardt A, Cauchemez B, Denjoy I, Jarry G, Mizon F, et al Decreased nocturnal standard deviation of averaged NN intervals. European heart journal. 2003;24(22):2061–2069. doi: 10.1016/j.ehj.2003.08.019 14613743 10.1016/j.ehj.2003.08.019

[14] Pierre B, Babuty D, Poret P, Giraudeau C, Marie O, Cosnay P, et al Abnormal nocturnal heart rate variability and QT dynamics in patients with Brugada syndrome. Pacing and clinical electrophysiology. 2007;30(s1):S188–S191. doi: 10.1111/j.1540-8159.2007.00635.x 17302702 10.1111/j.1540-8159.2007.00635.x

[15] Tokuyama T, Nakano Y, Awazu A, Uchimura-Makita Y, Fujiwra M, Watanabe Y, et al Deterioration of the circadian variation of heart rate variability in Brugada syndrome may contribute to the pathogenesis of ventricular fibrillation. Journal of cardiology. 2014;64(2):133–138. doi: 10.1016/j.jjcc.2013.12.001 24495503 10.1016/j.jjcc.2013.12.001

[16] Behar N, Petit B, Probst V, Sacher F, Kervio G, Mansourati J, et al Heart rate variability and repolarization characteristics in symptomatic and asymptomatic Brugada syndrome. Europace. 2016; p. euw224 doi: 10.1093/europace/euw224 10.1093/europace/euw224 27738060

[17] Kostopoulou A, Koutelou M, Theodorakis G, Theodorakos A, Livanis E, Maounis T, et al Disorders of the autonomic nervous system in patients with Brugada syndrome: a pilot study. Journal of cardiovascular electrophysiology. 2010;21(7):773–780. doi: 10.1111/j.1540-8167.2009.01702.x 20132392 10.1111/j.1540-8167.2009.01702.x

[18] Nakazawa K, Sakurai T, Takagi A, Kishi R, Osada K, Nanke T, et al Autonomic imbalance as a property of symptomatic Brugada syndrome. Circulation journal. 2003;67(6):511–514. doi: 10.1253/circj.67.511 12808268 10.1253/circj.67.511

[19] Imai K, Sato H, Hori M, Kusuoka H, Ozaki H, Yokoyama H, et al Vagally mediated heart rate recovery after exercise is accelerated in athletes but blunted in patients with chronic heart failure. Journal of the American College of Cardiology. 1994;24(6):1529–1535. doi: 10.1016/0735-1097(94)90150-3 7930286 10.1016/0735-1097(94)90150-3

[20] Savin WM, Davidson DM, Haskell WL. Autonomic contribution to heart rate recovery from exercise in humans. Journal of Applied Physiology. 1982;53(6):1572–1575. doi: 10.1152/jappl.1982.53.6.1572 7153152 10.1152/jappl.1982.53.6.1572

[21] Makimoto H, Nakagawa E, Takaki H, Yamada Y, Okamura H, Noda T, et al Augmented ST-segment elevation during recovery from exercise predicts cardiac events in patients with Brugada syndrome. Journal of the American College of Cardiology. 2010;56(19):1576–1584. doi: 10.1016/j.jacc.2010.06.033 21029874 10.1016/j.jacc.2010.06.033

[22] Amin AS, de Groot EA, Ruijter JM, Wilde AA, Tan HL. Exercise-Induced ECG Changes in Brugada Syndrome. Circulation: Arrhythmia and Electrophysiology. 2009;2(5):531–539. 19843921 10.1161/CIRCEP.109.862441

[23] Subramanian M, Prabhu MA, Harikrishnan MS, Shekhar SS, Pai PG, Natarajan K. The utility of exercise testing in risk stratification of asymptomatic patients with Type 1 Brugada pattern. Journal of cardiovascular electrophysiology. 2017;28(6):677–683. doi: 10.1111/jce.13205 28316113 10.1111/jce.13205

[24] Calvo M, Gomis P, Romero D, Le Rolle V, Béhar N, Mabo P, et al Heart rate complexity analysis in Brugada syndrome during physical stress testing. Physiological measurement. 2017;38(2):387 doi: 10.1088/1361-6579/aa513c 28134132 10.1088/1361-6579/aa513c

[25] Masrur S, Memon S, Thompson PD. Brugada syndrome, exercise, and exercise testing. Clinical cardiology. 2015;38(5):323–326. doi: 10.1002/clc.22386 25955277 10.1002/clc.22386 PMC6711014

[26] Lauer MS, Okin PM, Larson MG, Evans JC, Levy D. Impaired Heart Rate Response to Graded Exercise. Circulation. 1996;93(8):1520–1526. doi: 10.1161/01.CIR.93.8.1520 8608620 10.1161/01.cir.93.8.1520

[27] Gibbons RJ, Balady GJ, Bricker JT, Chaitman BR, Fletcher GF, Froelicher VF, et al ACC/AHA 2002 guideline update for exercise testing: summary article: a report of the American College of Cardiology/American Heart Association Task Force on Practice Guidelines (Committee to Update the 1997 Exercise Testing Guidelines). Journal of the American College of Cardiology. 2002;40(8):1531–1540. doi: 10.1016/S0735-1097(02)02164-2 12392846 10.1016/s0735-1097(02)02164-2

[28] Fox S 3rd, Haskell W. Physical activity and the prevention of coronary heart disease. Bulletin of the New York Academy of Medicine. 1968;44(8):950. 5243890 PMC1750298

[29] Dumont J, Hernandez AI, Carrault G. Improving ECG beats delineation with an evolutionary optimization process. IEEE Transactions on Biomedical Engineering. 2010;57(3):607–615. doi: 10.1109/TBME.2008.2002157 19171513 10.1109/TBME.2008.2002157

[30] Camm AJ, Malik M, Bigger J, Breithardt G, Cerutti S, Cohen R, et al Heart rate variability: standards of measurement, physiological interpretation and clinical use. Task Force of the European Society of Cardiology and the North American Society of Pacing and Electrophysiology. Circulation. 1996;93(5):1043–1065. doi: 10.1161/01.CIR.93.5.1043 8598068

[31] Auger F, Flandrin P, Gonçalvès P, Lemoine O. Time-Frequency Toolbox; 1996 CNRS France, Rice University.

[32] Orini M, Mainardi LT, Gil E, Laguna P, Bailón R. Dynamic assessment of spontaneous baroreflex sensitivity by means of time-frequency analysis using either RR or pulse interval variability. In: 2010 Annual International Conference of the IEEE Engineering in Medicine and Biology; 2010. p. 1630–1633. 10.1109/IEMBS.2010.5626877 21096388

[33] Hlawatsch F, Boudreaux-Bartels GF. Linear and quadratic time-frequency signal representations. IEEE signal processing magazine. 1992;9(2):21–67. doi: 10.1109/79.127284

[34] Costa AH, Boudreau-Bartels G. Design of time-frequency representations using a multiform, tiltable exponential kernel. IEEE Transactions on Signal Processing. 1995;43(10):2283–2301. doi: 10.1109/78.469860

[35] Bailón R, Laguna P, Mainardi L, Sornmo L. Analysis of heart rate variability using time-varying frequency bands based on respiratory frequency. In: Engineering in Medicine and Biology Society, 2007. EMBS 2007. 29th Annual International Conference of the IEEE; 2007. p. 6674–6677. 10.1109/IEMBS.2007.4353891 18003557

[36] Moody GB, Mark RG, Bump MA, Weinstein JS, Berman AD, Mietus JE, et al Clinical validation of the ECG-derived respiration (EDR) technique In: Computers in Cardiology. vol. 13; 1986 p. 507–510.

[37] Bailón R, Sornmo L, Laguna P. A robust method for ECG-based estimation of the respiratory frequency during stress testing. IEEE transactions on biomedical engineering. 2006;53(71273–1285). doi: 10.1109/TBME.2006.871888 16830932 10.1109/TBME.2006.871888

[38] Michael S, Graham KS, Davis GM. Cardiac Autonomic Responses during Exercise and Post-exercise Recovery Using Heart Rate Variability and Systolic Time Intervals—A Review. Frontiers in Physiology. 2017;8:301 doi: 10.3389/fphys.2017.00301 28611675 10.3389/fphys.2017.00301 PMC5447093

[39] He H, Bai Y, Garcia EA, Li S. ADASYN: Adaptive synthetic sampling approach for imbalanced learning. In: 2008 IEEE International Joint Conference on Neural Networks (IEEE World Congress on Computational Intelligence); 2008. p. 1322–1328.

[40] Robnik-Šikonja M, Kononenko I. Theoretical and empirical analysis of ReliefF and RReliefF. Machine learning. 2003;53(1–2):23–69. doi: 10.1023/A:1025667309714

[41] Guyon I, Elisseeff A. An introduction to variable and feature selection. Journal of machine learning research. 2003;3:1157–1182.

[42] Kohavi R, John GH. Wrappers for feature subset selection. Artificial intelligence. 1997;97(1):273–324. doi: 10.1016/S0004-3702(97)00043-X

[43] Kuncheva LI. Combining pattern classifiers: methods and algorithms. John Wiley & Sons; 2004.

[44] Mitchell JH. Neural circulatory control during exercise: early insights. Experimental Physiology. 2013;98(4):867–878. doi: 10.1113/expphysiol.2012.071001 23261851 10.1113/expphysiol.2012.071001

[45] Billman GE. The LF/HF ratio does not accurately measure cardiac sympatho-vagal balance. Frontiers in physiology. 2013;4:26 doi: 10.3389/fphys.2013.00026 23431279 10.3389/fphys.2013.00026 PMC3576706

[46] Kawazoe H, Nakano Y, Ochi H, Takagi M, Hayashi Y, Uchimura Y, et al Risk stratification of ventricular fibrillation in Brugada syndrome using noninvasive scoring methods. Heart Rhythm. 2016;13(10):1947–1954. doi: 10.1016/j.hrthm.2016.07.009 27424075 10.1016/j.hrthm.2016.07.009

[47] Romero D, Calvo M, Béhar N, Mabo P, Hernández A. Ensemble classifier based on linear discriminant analysis for distinguishing Brugada syndrome patients according to symptomatology. In: Computing in Cardiology Conference (CinC), 2016; 2016. p. 205–208.

